# Characterization of Changes in Key Odorants in Blueberries During Simulated Commercial Storage and Marketing by Sensory-Directed Flavor Analysis and Determination of Differences in Overall Perceived Aroma

**DOI:** 10.3390/foods14071244

**Published:** 2025-04-02

**Authors:** Fareeya Kulapichitr, Keith Cadwallader, David Obenland

**Affiliations:** 1 USDA, Agricultural Research Service, San Joaquin Valley Agricultural Sciences Center, 9611 South Riverbend Avenue, Parlier, CA 93648, USA; fareeya.kulapichitr@gmail.com; 2 Department of Food Science and Human Nutrition, University of Illinois at Champaign-Urbana, 1302 West Pennsylvania Avenue, Urbana, IL 61801, USA; cadwlldr@illinois.edu

**Keywords:** blueberry, storage, sensory evaluation, gas chromatography–olfactometry, stable isotope dilution analysis

## Abstract

To preserve quality and extend shelf-life, blueberries need to be maintained at low temperatures and high relative humidity during storage; however, during marketing, temperatures are considerably higher than what is optimal. The full impact of this varied temperature regime on flavor is unclear. Blueberries were stored at 1 °C for three weeks, followed by one week at 10 °C, and then two days at 20 °C, to simulate commercial conditions, and the aroma active compounds were evaluated. Gas chromatography–olfactometry combined with aroma extract dilution analysis and stable isotope dilution coupled with gas chromatography–mass spectrometry revealed that the key odorants of blueberries were affected by storage conditions, including 1-octen-3-ol, 1-octen-3-one, (Z)-3-hexenal, (E,Z)-2,6-nonadienal, and linalool. Extended storage at 1 °C resulted in a decrease in concentrations and odor activity values of most key odorants followed by their recovery as temperature increased. The perceived aroma from sensory testing confirmed the difference in the aroma of blueberries stored at 1 °C versus the control. The results indicated that commercial storage does not reduce blueberry aroma because blueberries are marketed at warmer temperatures and that blueberries should not be directly sold to consumers from cold storage.

## 1. Introduction

Southern highbush blueberry cultivars with a need for fewer chilling hours were developed in Florida and Georgia and provided the basis for a new industry in the Central Valley of California [1,2]. California is now one of the most important blueberry growing regions in the United States with a production value of more than USD 255 million in 2023 [3]. Due to its location, California exports the greatest amount of blueberries in the United States [4]. This is combined with increased domestic consumption during the last 15 years [3]. Blueberry fruits are highly perishable following harvest [5,6]; therefore, postharvest management is critical to supply demand for fresh blueberries in domestic and international markets [7].

Firmness, texture, color, flavor, sweetness, and juiciness are commonly utilized to determine blueberry eating quality [8]. Sugars and acids predominately determine sweetness and sourness, respectively, with moisture loss being a predominant factor in weight loss and fruit firmness [9]. For postharvest management, flavor maintenance is an important aspect of ensuring the quality of blueberries [6].

Volatile compounds in blueberries are responsible for the unique flavor and aroma of the fruit; they occur because of the interaction between dozens of aroma classes synthesized by the fruit during ripening and influenced by postharvest management [6,10]. Previous studies analyzing volatile compounds in blueberries by gas chromatography–mass spectrometry (GC-MS) and gas chromatography–olfactometry (GC-O) have shown that more than 100 volatile components have been identified, with a few being responsible for determining blueberry character [8,10]. It was also found that the volatile composition of blueberry fruit varies due to many factors, including the cultivar and environment [6,10].

To preserve quality and extend shelf-life, blueberries need to be maintained at refrigerated temperatures and high relative humidity during cold storage and transportation; however, during marketing and handling by consumers, blueberries are kept at much higher temperatures [11]. Previous research has been performed on blueberries to understand the impact of storage conditions in terms of time and temperature with regard to specific volatile compounds and sensory properties [6,12]. In the study by Yan et al. [6], which evaluated the effect of time and temperature on nine selected volatile compounds, the volatiles gradually declined during long-term storage at 0 °C and increased during subsequent storage at room temperature (25 °C) for up to 8 days. This later storage resulted in a quick deterioration in sensory acceptability that was associated with the increase in ethyl acetate, which imparts an over-ripe banana aroma; however, confirmation of this was not possible in the study. Sater et al. [12] investigated the change in sensory properties of six blueberry cultivars, including firmness, sweetness, sourness, blueberry flavor, green flavor, and off-flavors during cold storage. They pointed out that cold storage seems not to have a clear effect on generating significant off-flavors in any of the cultivars when the storage period was increased; however, a minty note was commented on for the perception of off-flavor in ‘Patrecia’ blueberries, and eucalyptol or methyl salicylate was surmised to be the cause for the off-flavor.

Although Yan et al. [6] made some progress in understanding the alterations in the aroma-active volatiles that may be involved in flavor changes during blueberry storage, their findings were based solely on odor activity values (OAVs) calculated using odor detection thresholds (ODTs) determined in water. However, based on the literature, an approach that relies only on OAVs is not sufficient to determine important odor characteristics due to the uncertainty in selected odor threshold values [13].

Therefore, a more detailed study using GC-O combined with OAV calculation is needed to screen and rank the character impact aroma compounds of blueberries and to fully understand the influence of storage on them, for which GC-O and OAV provide different yet complementary information [14]. GC-O analysis combined with dilution to threshold methods, such as aroma extracted dilution analysis (AEDA), is a valuable tool to help pre-screen odor-active compounds in a given food before the quantitation and determination of OAVs [15].

In addition, this study uses storage times and temperatures that could be possible during commercial cold storage, marketing, and purchase by the consumer to increase the utility of this study. Therefore, the aim of this study is to investigate the character impact of the odorants of blueberries, as influenced by different storage conditions. Sensory testing is also utilized to help confirm the effects of storage on overall aroma, as perceived by panelists. This research will provide an enhanced understanding of the effect of storage on blueberry flavor with the goal of improving blueberry quality for consumers.

## 2. Materials and Methods

### 2.1. Fruit

Highbush blueberries (Vaccinium corymbosum) ‘Snowchaser’, grown at the USDA Agricultural Research Service San Joaquin Valley Agricultural Sciences Center in Parlier, CA, were used for the experiments. No preharvest fungicide was applied. A harvest of 22 kg occurred on 18 May when the fruit reached commercial maturity, and 2 kg of the harvested samples (control; SC) were frozen immediately at −80 °C before further physicochemical analysis and sensory analysis. Meanwhile, the rest of the fruits were divided and stored in 170 g polyethylene clamshell boxes with openings for ventilation. The average blueberry size was 1.9 g. Commercial cold storage conditions were generated as follows: (1) 1 °C ± 0.5 °C for 3 weeks (S2), (2) 1 °C ± 0.5 °C for 3 weeks and 10 °C ± 0.5 °C for 1 week (S3), and (3) 1 °C ± 0.5 °C for 3 weeks, 10 °C ± 0.5 °C for 1 week, and removed to 20 °C ± 0.5 °C for 2 days (S4) to simulate typical commercial cold storage, distribution, and marketing. During cold storage at 1 °C and 10 °C, the relative humidity (RH) was at 85%. At each storage point, after sorting out decayed and damaged fruits, 2 kg of the blueberry samples were frozen in the same manner as the control for further analysis.

### 2.2. Determination of Weight Loss, Firmness, Total Soluble Solids, and Total Acidity

The blueberries to be evaluated at the final storage time point were weighed at the beginning of storage and at the end of storage to determine weight loss. Firmness was measured using twenty blueberries from each clamshell using a FirmTech FT7 (UP Umweltanallytische Producte GmbH, Ibbenburn, Germany). It was designed to measure the force required in g to cause a 1 mm deflection of a berry. Decayed or severely damaged blueberries were excluded from the measurements. A food homogenizer was used to juice the healthy berries, and the juice was then subjected to centrifugation at 8100× *g* for 10 min. From this juice, the soluble solids concentration (SSC) was measured using a digital refractometer (Atago, Tokyo, Japan), and titratable acidity (TA) was measured with an automatic titrator (Mettler model T50, Columbus, OH, USA). Except for weight loss, quality evaluation was performed on every clamshell. There were four replications performed for each of the quality determinations, except for weight loss, for which every clamshell was measured.

### 2.3. Sensory Evaluation Based on R-Index Analysis

The experimentation was given previous approval by the University of California Institutional Review Board (protocol 23014) on 20 October 2023.

The panelists were given an informed consent form to help them understand the nature of the experiment. If they then wanted to participate, they were asked to sign the form. Ground blueberry powders were prepared from the blueberries collected from individual storage treatments and compared for overall aroma with the samples prepared from SC using an R-index by the similarity ranking test [16]. John Brown’s computation [17] was used, which is based on the percentage of times a sample is ranked less than the control. A total of 30 g of frozen blueberries was ground with liquid N_2_ by an IKA analytical mill (IKA, Staufen, Germany); then, 20 g of fine blueberry powder was placed into a 125 mL PTFE wash bottle (Thermo Fisher Scientific, Fresno, CA, USA) modified for sniffing. The bottles were covered with foil to eliminate panelist bias and labeled with a 3-digit code. A set of 4 test samples (blueberry powders) was evaluated, including SC and another SC blueberry powder (blind control). Sensory evaluation for all the samples occurred within 4 h of preparation. Twenty-one staff members (five females and sixteen males; 18–61 years of age) participated in this test. The panelists were instructed to gently squeeze the bottle, sniff the air exiting the bottle, and rank the test samples on how similar they were to the control sample. The percentage R-index was compared to the critical value (n = 21) for a two-tailed test at α = 0.05 to determine a significant difference from the control.

### 2.4. Determination of Volatile Compounds

#### 2.4.1. Chemicals

All reagent chemicals were purchased from Sigma-Aldrich (St. Louis, MO, USA), with the following exceptions. Dichloromethane (DCM), methanol, hydrochloric acid (HCl), sodium hydroxide (NaOH, anhydrous), and sodium sulfate (Na_2_SO_4_, anhydrous) were bought from Thermo Fisher Scientific. Florisil was purchased from US Silica Company (Katy, TX, USA). Ultrahigh purity nitrogen and helium came from Linde Gas & Equipment (Fresno, CA, USA). Odorless water was prepared by boiling glass-distilled deionized water in an open flask to two-thirds of its original volume.

#### 2.4.2. Unlabeled Reference Standards

The purities of analytical standards were generally above 97%; they were checked by GC–flame ionization detection (GC-FID) for any potential oxidation or degradation. All reference standards were obtained from Sigma-Aldrich.

#### 2.4.3. Isotopically Labeled Standards

Isotope-labeled compounds, including hexanal-d_12_, 2-phenyl-d_5_-ethan-1,1,2,2-d_4_-ol, and linalool-d_3_, were purchased from CDN Isotopes (Pointe-Claire, Quebec, QC, Canada). Methyl-3-methylbutyrate-d_3_ was purchased from Santa Cruz Biotechnology (Dallas, TX, USA). Terpineol-d_3_ and eucalyptol-d_3_ were synthesized according to previously published procedures [18]. Meanwhile, ethyl-3-methylbutyrate-d_5_ was synthesized according to the previously published protocol of Zhu and Cadwallader (2019) [19].

#### 2.4.4. Aroma Extraction by Solvent-Assisted Flavor Evaporation (SAFE)

SAFE extraction is a process of solvent extraction to isolate volatiles at relatively low temperatures under high vacuum (10^−6^ bar), providing conditions that do not produce artifacts. Frozen blueberries (about 40 g) of each storage experiment (S1, S2, and S3) and SC were ground into fine powders with liquid nitrogen with an IKA analytical mill; then, 30 g of blueberry powder was weighed in a prechilled 250 mL Teflon FEP centrifuge bottle (Thermo Fisher Scientific) and dichloromethane (30 mL) was added. The bottles were sealed with Teflon FEP caps, shaken at 21× *g* with a New Brunswick Innova 2100 platform shaker (Eppendorf, Hauppauge, NY, USA) for 30 min, and then centrifuged at 1900× *g* for 15 min using an IEC HN-SII centrifuge (Damon/IEC Division, Needham, MA, USA). After the solvent layer was collected, the extraction was repeated 3 more times as above. The four dichloromethane extracts were combined, concentrated to 50 mL using a Vigreux column (approx. 20 cm) (40 °C), and stored at −80 °C until being subjected to solvent-assisted flavor evaporation (SAFE), as described previously [20]. The recovered extract was dried with sodium sulfate, concentrated with a Vigreux column to remove the bulk of the solvent, and then concentrated under a stream of nitrogen gas to 200 µL. The extracted volatiles were stored in 2 mL sample vials equipped with PTFE-lined caps at −80 °C until analysis.

#### 2.4.5. Gas Chromatography–Mass Spectrometry–Olfactometry (GC-MS-O)

GC-MS-O utilizes the human nose combined with mass spectrometry to identify volatiles that are odor-active. Analysis was conducted using an Agilent 7890 gas chromatograph equipped with an Agilent 5975 mass selective detector (Agilent Technologies, Inc., Santa Clara, CA, USA) and a sniffing port (ODP 4, Gerstel, Inc., Lithicum, MD, USA). Sample separations were performed using a DB-WAX column (30 m × 0.25 mm i.d., 0.5 μm film thickness, Agilent Technologies, Inc.) or HP-5 (30 m × 0.25 mm i.d., 0.25 μm film thickness, Agilent Technologies, Inc.).

A total of 2 µL of each extract was injected in the cold splitless mode using a Gerstel, CIS-4, PTV inlet (−50 °C initial temperature, 0.1 min delay, 12 °C/s ramp to 240 °C; 1.10 min splitless valve delay time). The initial temperature of the oven was 35 °C, and after 5 min, the temperature was increased by 10 °C/min to 225 °C, remaining at that temperature for 20 min. The flow rate of helium carrier gas was 1 mL/min. The mass spectra were recorded in full scan mode (35–350 a.m.u., scan rate 5.27 scans/s, interface temperature 240 °C, and ionization energy −70 eV). For GC–O, the flow was split 1:1 (*v*/*v*) between the MSD (240 °C) and ODP-4 (240 °C).

#### 2.4.6. Aroma Extract Dilution Analysis (AEDA)

GC-MS-O was performed by two experienced panelists to screen the aroma-active compounds by AEDA. This technique is useful in identifying which of the odorants are likely the most important in determining overall aroma. SAFE extracts were diluted stepwise with dichloromethane at a ratio of 1:2 *(v*/*v*), and each was analyzed by GC-MS-O until no odor was perceived. A flavor dilution (FD) factor is the highest dilution at which an odorant is last detected by GC-MS-O, i.e., if an odorant was detected in the 1:32 dilution but not the 1:64 dilution, it would be assigned an FD of 32.

#### 2.4.7. Compound Identification

The following criteria were used for compound identification: odor description agreement with an authentic standard (odor property recorded during GC-O), retention index (RI) match with the standard on two columns of different polarities, and mass spectral (MS) match with the standard. The RI of each compound was determined by comparison of the retention time to that of standard n-alkanes (C5–C30).

#### 2.4.8. Quantitation of Selected Volatile Compounds

##### Determination of Selected Volatile Compounds by SAFE-GC-MS

Nineteen selected key odorants with FDs ≥ 64 based on AEDA and/or previously reported as important volatile compounds associated with blueberry flavor [8,21] were quantitated by solvent extraction combined with SAFE-GC-MS, as described earlier. A solution of stable isotope-labeled internal standards (hexanal-d_12_, 2-phenyl-d_5_-ethan-1,1,2,2-d_4_-ol, and linalool-d_3_) was added to the sample prior to extraction to quantify these target odorants using stable isotope dilution analysis (SIDA). This method is highly accurate due to the similarity in the physicochemical properties between the isotopically labeled internal standards and the target analytes. Based on this approach, losses of the analytes caused by analytical procedures, i.e., extraction, distillation, or degradation, are compensated for. This differs from the use of generic internal standards that usually require the determination and use of recovery factors. Therefore, in this study, recovery factors were not calculated for compounds determined using unlabeled generic internal standards. Thus, these concentrations are considered semi-quantitative.

The target volatile compounds, isotopically labeled internal standards, selected MS ions, and response factors (R*f*) are indicated in Table S1.

A total of 2 µL of each blueberry volatile extract was analyzed using the aforementioned GC-MS-O system under the same analytical conditions, except that selective ion monitoring (SIM) (dwell time, 50 ms; ions monitored) was used for quantitation. The unique quantitation mass ion was chosen to ensure the highest response and lowest interference (Table S1).

##### Determination of Selected Volatile Compounds by Headspace Solid-Phase Microextraction–Gas Chromatography–Mass Spectrometry (HS-SPME-GC-MS)

HS-SPME-GC-MS was used to determine another four selected key aroma compounds, i.e., methyl-3-methylbutanoate, ethyl-3-methylbutanoate, eucalyptol, and α-terpineol, with the same criteria as those in the earlier section for better sensitivity than SAFE-GC-MS extraction, as previously described [8]. With SPME, volatiles in a sample are adsorbed onto a coated fiber and then desorbed into a hot GC inlet. A total of 30 g of frozen blueberries was powdered with liquid nitrogen by an IKA analytical mill. Then, 2 g of blueberry powder was weighed into a prechilled 20 mL vial and mixed with 5 mL of citrate buffer (0.2 M, pH 3.2, 1% NaF, saturated with NaCl). Then, appropriate isotope-labeled internal standards (d_3_-methyl 3-methylbutanoate, d_5_-ethyl 3-methylbutanoate, d_3_-eucalyptol, and d_3_-α-terpineol) were added, and the vial was sealed with a PTFE-lined septum cap. Triplicate analyses were performed on each sample.

Analysis was performed with a 7890 GC-MS system (Agilent Technologies, Inc.) coupled to a time-of-flight mass spectrometer (TOF-MS) (Pegasus 4D; LECO^®^; St. Joseph, MI, USA) with a CombiPal (Leap Technologies, Inc., Fort Lauderdale, FL, USA) automated injector. Prior to the run, the sample was incubated (45 °C, 250 rpm) for 10 min prior to sampling. Following this, an SPME fiber (Supelco, 2 cm, DVB/Carboxen/PDMS) was deployed into the sample vial headspace and incubated for 50 min at 45 °C prior to the fiber being removed from the sample vial and desorbed for 20 min into the 260 °C splitless inlet of the GC. Volatile separation was accomplished with a Stabilwax column (30 m × 0.25 × 0.25 µm) manufactured by Restek (Bellefonte, PA, USA), with helium carrier gas at a flow rate of 1 mL/min. The oven temperature was ramped from 40 °C to 225 °C at a rate of 4 °C per minute. The MS interface was 230 °C, and the ionization energy was 70 eV, with a 35 to 300 amu mass range and a scan rate of 50 scans/s.

#### 2.4.9. Calculation of Amounts of Selected Volatile Compounds

Peak areas for selected ions of the target analytes and internal standards were determined using Agilent Enhanced Chemstation E.02 (Agilent Technologies, Inc.) for SAFE extraction and Leco Chroma TOF software (version 3.34) for SPME analysis. Concentrations were calculated as follows:

(1) $${\mathrm{Concentration}}_{t}(µg/\mathrm{kg})={\mathrm{mass}}_{i}(µg)\;\times R_{f}\;\times \frac{\left[\mathrm{peak}\;\mathrm{area}\;\mathrm{ratio}\right]}{[\mathrm{sample}\;\mathrm{amounts}\;(\mathrm{kg})]}\;\;$$

where t is the target analyte, mass_i_ is the mass of the labeled internal standard added to the sample prior to analysis, peak area ratio is the ratio between the peak area of target analyte and the peak area of the labeled internal standard, and R*_f_* is the response factor. The R*_f_* for a target analyte consisted of the inverse of the slope of the calibration plot of the area ratio versus the mass ratio and utilized five levels of standard (unlabeled) against the labeled internal standard.

#### 2.4.10. Determination of Odor Activity Values (OAVs)

The odor activity value (OAV) of a compound was calculated by dividing its concentration in the blueberry samples by their published ODTs in water [22].

### 2.5. Statistical Analysis

Mean values from physicochemical analyses were analyzed for significant differences at *p* ≤ 0.05 by one-way ANOVA and Tukey’s test for mean separations. The statistical analyses utilized SPSS version 13.0 (SPSS Inc., Chicago, IL, USA). Multivariate analysis of the significant key volatiles for the four types of blueberry samples was performed by principal component analysis (PCA) using XLStat v.2018.2.10 (Lumivero, Denver, CO, USA). This examined the distribution of the 23 significant key volatiles with FDs ≥ 64, and/or previously reported as important volatile compounds associated with blueberry flavor [8,21], determined from univariate analysis (one-way ANOVA) for the four blueberry treatments.

## 3. Results and Discussion

### 3.1. Effect of Storage Conditions on Total Acidity, Firmness, and Soluble Solids in Blueberries

Total acidity, soluble solids, weight loss, and firmness were determined to understand the effect of the storage treatments on basic quality parameters (Table 1). No change was observed in soluble solids, while total acidity only differed between SC and S2. Total weight loss increased in a linear manner until S3, where weight loss greatly increased (Figure S1). Firmness tended to decrease during storage, from SC to S3, though only the firmness of S3 significantly differed among the storage treatments. Loss of firmness is a common feature during blueberry storage; it is closely linked to weight loss, except during low weight loss, when blueberry firmness often temporarily increases [9,23]. As found in this study, firmness loss in blueberries is also temperature-dependent, with greater rates of firmness loss occurring at warm versus cold temperatures. Firmness is very important to blueberry quality, as blueberries that are perceived to be soft are not as well-liked by consumers [2].

### 3.2. Effect of Storage Conditions on Character Impact Odorants of Blueberries

#### 3.2.1. AEDA Analysis by GC-MS-O

GC-MS-O is a powerful technique that utilizes both human and mass spectrometry detectors to identify key odorants based on odor characteristics and mass spectra, and it is widely used to study food aromas and other applications as well [24]. In this study, we utilized SAFE extraction followed by GC-MS-O and AEDA to identify the odor potency of volatiles extracted from SC and the three storage treatments. Overall, it was found that most key odorants tended to have reduced flavor dilution factors (FDs) due to cold storage (S1) relative to SC, whereas there was a recovery (increase in FDs) following cold storage as storage progressed with the warmer storage treatments (S2 and S3) (Table 2). Among the key odorants were 4 esters, 7 aldehydes, 4 alcohols, 4 ketones, 5 terpenes, and 10 miscellaneous compounds that contributed to the impression of overall blueberry aroma with FDs ≥ 32–64.

##### Esters

Esters are formed from carboxylic acids and alcohols in fruits during ripening and are responsible for the fruity note of blueberries [8]. Methyl 2/3-methylbutyrate and ethyl 2/3-methylbutyrate were identified in this study. Ethyl 3-methylbutyrate had the highest FD (4096, 1024) in SC, which decreased dramatically in S1 (8, 4) and then rose in both S2 and S3 (64, 32). On the other hand, ethyl-2-methylbutytate was detected at the highest FD in S1 (128), followed by SC and S3, while it was not detected in S2. Methyl 2-methylbutyrate and methyl 3-methylbutyrate had equal FDs (1024) in SC; their FDs decreased in S1 (128 and 64), and then both further declined in S2 (8 and 64, respectively) followed by an increase in S3. Previous research reported the importance of methyl 2/3-methylbutanoate and ethyl 2/3-methylbutanoate in ‘Bluecrop’ and ‘Elliott’ blueberries, which had high FDs at 128–512 [8], in agreement with our study. Ethyl 2/3-methylbutanoate was not previously identified in ‘Snowchaser’ blueberries [21], which could be due to the fact that the panelists in the SPME-GC-O study could not perceive the esters due to their low concentrations [8]. In addition, the difference could be due to this study, as SAFE extraction was used to extract, concentrate, and discriminate trace volatiles from the artifacts coupled with enrichment by cold-splitless injection.

##### Aldehydes and Corresponding Alcohols: Lipid Oxidation Products from the Lipoxygenase Pathway

C6 aldehydes and their corresponding alcohols that contribute characteristic “green-leaf” and “fresh fruity” notes are derived from linoleic and α-linolenic acids in the membrane lipids of blueberries [8]. The synthesis of these aldehydes occurs via the oxylipin pathway using multiple enzymatic reactions, such as those catalyzed by lipoxygenases and hydroperoxide lyase. The aldehydes may be converted to their corresponding alcohols by alcohol dehydrogenases [25]. Aldehydes could also be reduced to their corresponding alcohols by aldehyde reductases or oxidized to C6 acids [8].

The results of AEDA confirmed that hexanal, (*Z*)-3-hexenal, (*E*)-2-hexenal, (*E*)-3-hexen-1-ol, and (*Z*)-3-hexen-1-ol provide fresh berry and green aroma notes to the blueberry samples. Their FDs showed a similar dramatically decreasing trend relative to SC when the blueberries were subjected to the S1 treatment. The odor potential was impressively enhanced in the S2 and S3 treatments for (*Z*)-3-hexenal (FD = 16,384 and 4096), but hexanal and (*E*)-2-hexenal did not increase from S1 to S2. This pattern, in terms of an inhibitory impact of S1 on FDs, was also repeated for the alcohol-derived volatiles (*E*)-3-hexen-1-ol and (*Z*)-3-hexen-1-ol, with mixed effects on the FDs of S2 and S3.

Other important aldehydes with moderate odor potentials were 3-methylbutanal and (*E*,*Z*)-2,6-nonadienal, which were previously identified in blueberries [8,26,27] and are reported as having malty [28] and green and cucumber notes [8], respectively. 3-methylbutanal was reported to be synthesized by the decomposition of by-products during the biosynthesis of valine, leucine, and isoleucine [28]. Meanwhile, 2,6-nonadienal is generated from polyunsaturated carboxylic acids [8,29]. Unlike the other aldehydes, both 3-methylbutanal and (*E*,*Z*)-2,6-nonadienal increased from SC to S1. For 3-methylbutanal, the FDs continued to increase throughout storage. Conversely, for (*E*,*Z*)-2,6-nonadienal, the FDs soared to 16,384 and 1024 in the S1 blueberry samples then decreased in S2 (8192, 64) and S3 (512, 128).

The general overall decrease during the initial cold storage in the major aldehydes and alcohols (e.g., (*E*)-3-hexen-1-ol, and (*Z*)-3-hexen-1-ol, hexanal, (E)-2-hexenal, (Z)-3-hexenal, and (*E*,*Z*)-2,6-nonadienal), which are products of the lipoxygenase pathway (S1), is consistent with previous studies [6,30]. This has also been observed in peaches, tomatoes, and apples [25,31,32]. Chilling could possibly inhibit the C6 aldehyde and alcohol production due to decreases in hydroperoxide lyase and alcohol dehydrogenase activities in the oxylipin pathway [25]. In the same study, it was found that warming tomatoes at 20 °C for up to 4 days after chilling was found to increase the regulation of a group of genes that controlled lipoxygenase activity and slow down the expression of other genes, which may explain the recovery of volatiles at warmer temperatures (S2 and S3 in this study).

##### Terpenes: Products of Terpene Synthase

Terpenes and their derivatives are generally formed enzymatically from acetyl CoA and pyruvate provided from carbohydrate substrates via the mevalonate pathway in plastids and the cytoplasm of plants [8,33]. These compounds are responsible for the formation of membrane structure, photosynthesis, redox chemistry, growth regulation, plant defense, and the response to stress [33,34].

Eucalyptol, linalool, α-terpineol, citronellol, nerol, geraniol, and geranyl acetone were found to have odor potentials with FDs ≥ 64 in this study. With the exception of nerol and linalool, the predominant FD change in S1 relative to SC was to increase or stay the same. Meanwhile, when the blueberries were subjected to S2, the FDs stayed the same or declined, except for large increases in the FDs of α-terpineol and nerol. However, an impressive enhancement in the FDs was indicated for linalool (16,384) and geraniol (8192) following S3.

Among these terpenes, the highest FDs were observed for linalool, nerol, and geraniol, which peaked at 16,384. This is in agreement with previous research that demonstrated that these odorants had large FDs in ‘Bluecrop’ and ‘Elliot’ blueberries relative to the others identified [8]. Generally, linalool and geraniol have been noted to make significant aroma contributions to highbush blueberry [8,35,36].

A decline in terpenes during cold storage of fruits has been commonly reported in blueberries [6,30], muscat grapes [37,38], and peaches [32]. It was proposed that in grapes, this loss was due to emission from the berry surface during cold storage [38,39]. An increase in terpenoids, as was seen in this study with linalool, can occur when fruits are warmed after cold storage, which was demonstrated to occur in peaches [32] and muscat grapes [38], although the authors did not propose a reason for this. Eucalyptol is responsible for the minty note in blueberries, and its odor importance (high FDs) agrees with previous research on ‘Bluecrop’ blueberries [8].

##### Benzenoids and Phenolic Compounds: Derived Products of Phenylalanine

Benzenoid and phenylpropanoid volatile compounds are derived primarily from phenylalanine involving several enzymes for their synthesis. They contribute to the aromas and scents of plants and are involved in plant communication with the environment [33,34,40,41]. In this study, guaiacol, 4-vinylguaiacol, m-cresol, eugenol, (Z)-isoeugenol, and vanillin showed potential as key odorants with FDs ≥ 64. Clove, smoky, phenolic, and spicy sensory notes are delivered by guaiacols, phenols, and cresols in a large variety of foods and beverages [42]. In this research, the FD of guaiacol increased from SC (16, 8) to S1 (1024); then, it gradually decreased until the end of storage. The highest FD for eugenol was SC, with lesser values throughout storage. The patterns in FDs for 4-vinylguaiacol, (Z)-isoeugenol, and vanillin varied, with peaks occurring in S2 and in SC for vanillin.

Guaiacol has been previously detected in blueberries [43], but its odor potential based on AEDA and FDs was reported in this present study for the first time, as well as the detection of 4-vinylguaiacol and its odor potency in blueberries. The cause of the changes in FDs during storage is unclear, but it is likely tied to how the berry’s metabolism and enzymatic reactions were altered by storage. To our knowledge, this was the first time that these two odorants were detected in ‘Snowchaser’ blueberries.

Eugenol is a cinnamate derivative commonly found in clove oil [44], which is responsible for clove-like notes; meanwhile, vanillin contributes to the vanilla note and is a well-known component of vanilla beans [45]. Both eugenol and vanillin have been reported to contribute to character-impact odorants of the ‘Bluecrop’ and ‘Elliot’ [8] and bog blueberry varieties in previous research [45].

In this study, eugenol showed the tendency to gradually decrease in cold storage from S1 to S2; then, the aroma potency was restored in S3. Whereas for vanillin, a pattern of fluctuation was indicated from the decrease in FD in SC (1024 and 256) to 128 in S1, then a restored S2 level, and, finally, the decrease in S3 (128).

Other minor phenol derivatives responsible for phenolic, metallic, and leather aromas in blueberries have been previously reported [8,21]. In the present study, m-cresol and p-cresol were found to have low odor potencies, except for m-cresol in S3. In a previous study, FDs of phenylacetaldehyde (rosy note), cinnamaldehyde (cinnamon note), and cinnamyl alcohol (rosy note) ranged from 2 to 64 in wild and lowbush blueberries [45] and in highbush blueberries [8], which agree with results of the present study with respect to cinnamyl alcohol.

##### Miscellaneous Compounds

1-Octen-3-one and 1-octen-3-ol had previously been reported for their contributions to the mushroom note of blueberries, while methional contributes to the cooked potato note [8,21]. Sotolon has been widely noted for providing a seasoning note in food [28] and may be formed by chemical or biochemical means [46]. Sotolon was identified for the first time in blueberries in this study. Other miscellaneous compounds were 2,3-butanedione (buttery note), 2-heptanone (green note), and γ-decalactone (coconut note). The odor potential of 1-octen-3-one was very high throughout storage, peaking in S2 (16,384 and 8192), while 1-octen-3-ol had the highest FD in S1 (16,384; Wax column) and decreased in later storage treatments. The FDs of these two volatiles were higher than those reported in previous research with ‘Bluecrop’ highbush blueberries (FDs of 128 and 256); however, the high relative contribution of these odorants agrees with this study. γ-Decalactone also showed a high potential aroma contribution in SC (FD = 4096, 2048), but this decreased as storage progressed. Meanwhile, for 2,3-butanedione, the FD values were relatively low, with the highest FD occurring in S1.

The identification of 3-methylbutyric acid, responsible for a cheesy note, was in accordance with previous GC-O work on blueberry aroma in the ‘Bluecrop’ and ‘Elliot’ varieties (FD = 32 and 16, respectively) [8]. In this study, the FD for SC was 32, which declined in S1 and fluctuated thereafter in later storage.

Even though β-damascenone, a norisoprenoic ketone, was identified as having a honey note in this study, the FDs detected were quite low (1–16), showing the lower contribution of this compound to the overall impression of blueberry aroma. In previous studies, Qian et al. (2021) indicated that this compound had an impact on ‘Bluecrop’ and ‘Elliot’ blueberry flavor, with FDs of 64–256 [8].

#### 3.2.2. Quantitation of Selected Key Odorants and OAVs

FDs from GC-O and AEDA can aid in the ranking of potent odorants with respect to their relative importance in a type of food [47]. However, with this type of analysis, extraction efficiencies and matrix influences are not accounted for [48]. This means that FDs are not a direct measure of an odorant’s actual contribution; thus, odor activity values (OAVs; the ratio of concentration to its ODT) should be considered in addition to FDs to better gauge the importance of an odorant. Among the 18 odorants selected for quantitation in this study, (E,Z)-2,6-nonadienal had the highest OAV of 1962 in the SC blueberries, which decreased in S1 (10) and S2 (145) and then substantially increased to 8923 in S3 (Table 3). The second most important character-impact odorant based on its OAVs was (Z)-3-hexenal, which continuously increased from SC (OAV = 135) to S3 (OAV = 396). The production of unsaturated aldehydes from the oxidation of linolenic acid could be formed during sample preparation and volatile extraction if blueberry samples are prepared by maceration, although the addition of salt could inhibit the enzyme activity [26]. (Z)-3-Hexenal is thermally unstable with heat and is easily isomerized to (E)-2-hexenal or degraded to its corresponding alcohols, such as (Z)-3-hexen-1-ol, via enzymatic conversion, which can occur during aroma isolation and analyses [14,49]. Therefore, in this study, the grinding of samples with liquid nitrogen and the application of cold-splitless PTV injection were applied with the aim of minimizing these transformations and loss of other volatiles since high excessive temperatures in the inlet could provoke the decomposition of labile sample constituents [50]. This possibly correlates with the lower content of (E)-2-hexenal (21.64–109.72 µg/kg) and (Z)-3-hexen-1-ol (8.18–26.56 µg/kg) in this study compared to previous studies that used SPME analysis [6,26]. Other volatiles that also had a high impact on the overall aroma based on their OAVs were 1-octen-3-one and 1-octen-3-ol. They both had lower OAVs in S1 relative to SC and then increased again in S3. Among terpenes, linalool had the highest OAV in SC (113) and then declined dramatically in S1 (37). The range of concentrations agreed well with values reported in ‘Snowchaser’ blueberries (84–98 µg/kg) [26] and other varieties of blueberries, including ‘Duke’, ‘Draper’, ‘Bluecrop’, ‘Calypso’, ‘Elliott’, and ‘Last Call’ (1198–34,201 µg/kg) [43]. The negative impact of cold storage (S1) on the linalool concentration and later recovery at warmer temperatures (S2 and S3) was also observed in blueberries by Yan et al. [6]. Eugenol imparts a clove-like note and was reported to be an aroma-active component of fresh blueberry juice [51]. In this study, the OAV for eugenol ranged from 4 to 11 and increased from S1 after the storage temperature increased. However, the range (5.15–14.88 µg/kg) of eugenol in this experiment was higher than reported for rabbiteye blueberries (~1.5 µg/kg) [6] and lower than ‘Bluecrop’ and ‘Elliot’ (58.7 µg/kg and 92.4 µg/kg, respectively), which is possibly due to the difference in variety, storage conditions, and manner of sample preparation. For eucalyptol, the OAV (30–50) did not have a consistent pattern in response to storage. The concentrations of eucalyptol in this research (32.53–55.55 µg/kg) agree with values reported for ‘Snowchaser’ (40–46 µg/kg) [26].

With respect to the esters, i.e., methyl 3-methylbutyrate and ethyl 3-methylbutyrate, their concentrations and OAVs were affected differently by the storage conditions. Methyl 3-methylbutyrate changed in concentration from 9.21 µg/kg to 22.33 µg/kg from SC to S1, then decreased in S2 (6.79 µg/kg), and, finally, increased slightly in SW3 (15.5 µg/kg). On the contrary, the concentration and the OAVs of ethyl 3-methylbutyrate exhibited little change. Their concentrations were within the expected concentration range reported for ‘Snowchaser’ (Methyl 3-methylbutyrate: 39–128 µg/kg and ethyl 3-methylbutyrate: 0–8 µg/kg) as determined by SPME [26].

Regarding hexanal, one of the green aromas, the concentration dramatically dropped down from 84.09 µg/kg in SC to 9.72 µg/kg in S1; then, it slightly increased in S2 (10.82 µg/kg) and built up to 16.74 µg/kg, which agreed with the study by Yan et al. [6], who observed the same cold versus warm storage effect with rabbiteye blueberries. The values in SC were within the previous range of ‘Snowchaser’ (66–198 µg/kg) [26].

For the lignin-derived compounds, i.e., vanillin and guaiacol, despite their high FDs, their OAVs were relatively low. Vanillin remained relatively similar across treatments, while guaiacol increased in both measures at the highest temperature of storage (S3).

Disparities exist between the changes in FDs and OAVs from SC throughout storage and the degree of change for some odorants in this present study (Table 3 and Table 4). FD factors provide guides to which compounds are possibly odor-important. They are generally not considered absolute because they do not consider recovery factors in the analysis. On the other hand, OAVs are calculated based on accurate concentrations and published odor detection thresholds. Imperfect relationships between FDs and OAVs have been mentioned in prior blueberry aroma research [8] and other research as well [52]. Regarding previous research, AEDA uses air as the matrix, whereas OAVs are calculated from the odor thresholds in water, leading to differences in the results [14,53]. Various studies have critically compared different OAV calculation methodologies, based on either the ODT in water or OTD in actual food matrices, with the variation in the results being due to the different principles applied, each subject to methodology advantages and limitations. The calculation of OAVs relies on measuring the concentration accurately and on the determined odor threshold in the food matrix [54], as a relatively high ODT and food matrix affect aroma release [48,55]. Therefore, there is no universal standard method or technique to determine the relative importance of odorants. Additionally, sometimes, using different analytical approaches combined with GC-O will enable more information and reduce the errors associated with the use of only a single method [15]. The loss of concentrations in key aroma volatiles due to cold storage found in this study was also observed in prior studies with tomatoes, apples, and peaches [25,31,32,56].

If blueberries or other fruits were to be consumed immediately after cold storage, there would likely be a loss in aroma and a potential loss in flavor due to the loss of important volatiles. However, fruit briefly held at a warmer temperature prior to being sold is generally part of the commercial marketing process and, as indicated in this study, only a short amount of time is required at either 10 °C or, preferably, 20 °C to recover some of the key odorants in blueberries. Many volatiles in fruit that were previously depleted during cold storage will recover at warm temperatures, with the potential reasons for this being the regaining of enzymatic activity needed for volatile synthesis [25] and the availability of volatile precursors that accumulated during cold storage [31]. It needs to be acknowledged, however, that we used commercial packaging and that other types of packaging could potentially alter the relationship between volatiles and temperature that we observed.

**Table 3:** Retention indices (RIs), concentrations, and odor activity values (OAVs) of selected potent odorants in blueberries stored with different conditions based on a Wax column.

Target Odorants	RI	Odor Threshold (µg/L) ^2^	Concentrations (µg/kg) ^1^				OAVs			
			Storage Treatments				Storage Treatments			
			SC	S1	S2	S3	SC	S1	S2	S3
Methyl-3-methylbutyrate	1022	4.4	9.21 ± 0.96 ^C^	22.33 ± 1.53 ^A^	6.79 ± 0.94 ^C^	15.5 ± 0.89 ^B^	2	5	2	4
Ethyl-3-methylbutyrate	1072	0.11	1.09 ± 0.04 ^A^	0.98 ± 0.05 ^B^	1.04 ± 0.03 ^AB^	0.99 ± 0.02 ^AB^	10	9	9	9
Hexanal	1083	10	84.09 ± 1.76 ^A^	9.72 ±1.31 ^B^	10.82 ± 1.59 ^BC^	16.74 ± 1.92 ^CD^	8	1	1	2
(*Z*)-3-Hexenal	1145	0.21	28.38 ± 1.36 ^C^	38.39 ± 1.75 ^B^	42.43 ± 3.27 ^B^	83.14 ± 0.78 ^A^	135	183	202	396
Eucalyptol	1210	1.1	35.77 ± 2.05 ^B^	55.5 ± 1.13 ^A^	32.53 ± 1.29 ^B^	50.73 ± 1.33 ^A^	33	50	30	46
(*E*)-2-Hexenal	1221	190	109.72 ± 10.65 ^A^	21.64 ± 4.38 ^B^	27.02 ± 1.91 ^B^	41.77 ± 4.31 ^B^	1	<1	<1	<1
1-Octen-3-one	1278	0.036	13.73 ± 1.51 ^A^	0.68 ± 0.14 ^C^	5.34 ± 1.34 ^BC^	10.45 ± 1.88 ^AB^	381	19	148	290
(*E*)-3-Hexen-1-ol	1374	110	10.27 ± 0.53 ^A^	5.05 ± 0.08 ^D^	8.55 ± 0.21 ^B^	7.09 ± 0.15 ^C^	<1	<1	<1	<1
(*Z*)-3-Hexen-1-ol	1387	13	8.18 ± 0.04 ^C^	15.08 ± 0.41 ^B^	26.56 ± 0.82 ^A^	11.26 ± 0.48 ^BC^	1	1	2	1
1-Octen-3-ol	1453	1	53.81 ± 4.51 ^B^	2.15 ± 0.10 ^B^	4.76 ± 0.31 ^B^	266.97 ± 30.70 ^A^	54	2	5	267
Linalool	1550	6	678.16 ± 17.95 ^A^	221.02 ± 7.71 ^C^	364.21 ± 14.26 ^B^	267.47 ± 14.88 ^C^	113	37	61	45
(*E*,*Z*)-2,6-Nonadienal	1587	0.01	19.62 ± 0.24 ^B^	0.10 ± 0.01 ^D^	1.45 ± 0.35 ^C^	89.23 ± 0.58 ^A^	1962	10	145	8923
3-Methylbutyric acid	1678	132	63.06 ± 4.05 ^A^	25.52 ± 0.17 ^B^	29.13 ± 0.29 ^B^	21.99 ± 2.12 ^B^	<1	<1	<1	<1
α-Terpineol	1701	330	82.17 ± 12.37 ^A^	82.57 ± 6.89 ^A^	59.2 ± 12.03 ^A^	92.43 ± 21.75 ^A^	<1	<1	<1	<1
Citronellol	1784	40	0.74 ± 0.01 ^C^	0.002 ± 0.00 ^C^	2.26 ± 0.34 ^B^	287.41 ± 11.76 ^A^	<1	<1	<1	<1
Nerol	1802	300	6.93 ± 0.03 ^B^	0.02 ± 0.00 ^B^	4.44 ± 0.04 ^B^	236.94 ± 15.93 ^A^	<1	<1	<1	<1
Guaiacol	1861	2.5	2.11 ± 0.08 ^B^	0.005 ± 0.00 ^B^	0.60 ± 0.15 ^B^	15.92 ± 1.67 ^A^	1	<1	<1	6
Geraniol	1867	5	3.75 ± 0.56 ^B^	0.01 ± 0.00 ^B^	2.60 ± 0.03 ^B^	48.38 ± 8.94 ^A^	1	<1	1	10
(*E*)-Cinnamaldehyde	2038	790	0.15 ± 0.03 ^B^	0.01 ± 0.00 ^B^	0.28 ± 0.02 ^B^	4.35 ± 1.09 ^A^	<1	<1	<1	<1
*γ*-Decalactone	2165	11	0.24 ± 0.00 ^C^	0.01 ± 0.00 ^D^	0.81 ± 0.01 ^B^	2.21 ± 0.08 ^A^	<1	<1	<1	<1
Eugenol	2171	1.3	5.15 ± 0.00 ^C^	5.28 ± 0.13 ^C^	14.88 ± 0.29 ^A^	11.88 ± 0.27 ^B^	4	4	11	9
4-Vinylguaiacol	2187	20	0.06 ± 0.01 ^C^	0.01 ± 0.00 ^C^	1.50 ± 0.20 ^B^	3.19 ± 0.17 ^A^	<1	<1	<1	<1
Vanillin	2569	25	57.27 ± 0.46 ^B^	29.36 ± 2.26 ^C^	82.39 ± 2.23 ^A^	57.89 ± 2.66 ^B^	2	1	3	2

^1^ Concentration was calculated as mean ± S.D. (µg/kg). Duplicate based on a DB-WAX column. Different letters within a row are significant at *p* ≤ 0.05. ^2^ Odor detection threshold values in water (µg/L) were obtained from [8,16,57,58].

**Table 4:** R-index values based on the degree of similarity ranking versus the control (SC) of the overall aroma of blueberry powders prepared from blueberries stored under different conditions.

Storage Treatments	R-Index JB ^a^
S1	81 *
S2	57
S3	62

^a^ Calculated using John Brown’s computation against the SC blueberry sample (control) expressed as a percent. There were 5 females and 16 males (18–61 years old). Critical R-index value = 66.44 using a two-tailed test. * Significantly different from the control (SC) at α = 0.05.

### 3.3. Effects of Storage Conditions on the Overall Perceived Aroma of Blueberries

The R-index by ranking (degree of aroma similarity) sensory evaluation was conducted to determine if changes in the concentration of aroma compounds found in S1, S2, and S3, relative to SC, could be perceived by the panelists. It should be acknowledged that the number of panelists and the imbalance in gender are not optimal for this type of sensory testing. The results (Table 4) indicated that only S1 significantly differed from SC (R-index value = 81), whereas S2 and S3 were not significantly different than SC (R-index = 57 and 62, respectively). The dissimilarity in aroma perception between S1 and SC could be due to the significant reduction in (*E*,*Z*)-2,6-nonadienal, 1-octen-3-ol, 1-octen-3-one, and linalool, considering that these key odorants declined from SC to S1 and had the highest OAVs (Table 3). Additionally, the increase in eucalyptol in S1 (OAV = 50) may have played some role in the altered aroma since minty perception was commented on in relation to off-flavor in ‘Patrecia’ blueberries [12]. However, Yan et al. (2020) did not observe an objectionable off-flavor in blueberries stored at 0 °C for 60 d but did note a change in freshness compared to 15 d storage [6]. In comparison to S1, the similarity in S2 and S3 to the control is likely due to the restoration of key odorants that declined in S1.

### 3.4. Principal Component Analysis (PCA)

**Figure 1:**
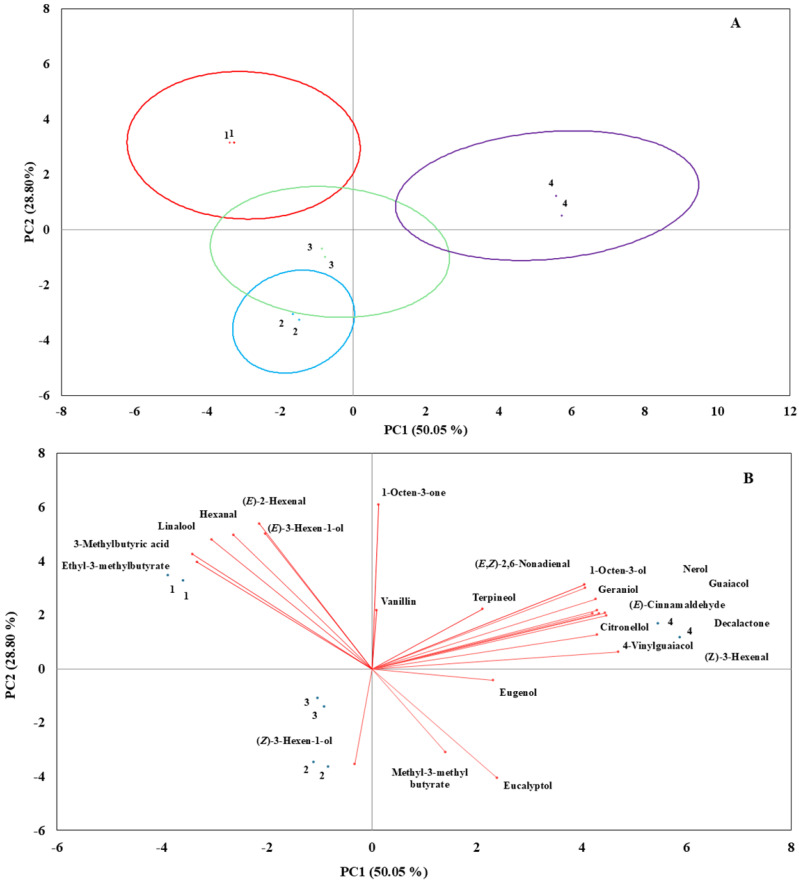
Effect of storage condition on 20 significant key odorants from the analysis using blueberries immediately after harvest (SC) and after 3 weeks of storage at 1 °C (S1), followed by a further 1 week at 10 °C (S2) and then 2 d at 20 °C (S3). (**A**) PCA score plot; (**B**) PCA biplot. Numbers within the graph are 1: SC, 2: S1, 3: S2, and 4: S3.

To further examine the changes in blueberry aroma during storage and simulated marketing, the concentration results for 23 selected key odorants were subjected to PCA. The PCA score plot shown in Figure 1A was responsible for an overall cumulative variance of 78.85%. The score plot was able to clearly separate and cluster SC from S1 based primarily on the concentrations of ethyl 3-methylbutyrate, 3-methylbutyric acid, linalool, and hexanal (Figure 1B). This loss of aroma compounds in S1 agreed with the R-index results (Table 4). In addition, S3 clearly separated from SC and S1 due to the large accumulation of (*E*,*Z*)-2,6-nonadienal, 1-octen-3-ol, guaicol, (*E*)-cinnamaldehyde, decalactone, geraniol, nerol, citronellol, 4-vinylguaiacol, and (*Z*)-3-hexenal that occurred as the blueberries were warmed to 20 °C (Figure 1B).

## 4. Conclusions

This research is the first to examine the impact of storage on blueberry aroma by comparing overall perceived aroma characteristics and aroma-active components using GC-O in combination with the quantitation and calculation of OAVs. An important part of this study was also that the storage conditions were commercially relevant. These aspects of this research greatly add to the work by Yan et al. [6], who conducted the most similar research to this study, examining storage effects on blueberry volatiles. GC-O analysis based on AEDA indicated two new volatiles, i.e., sotolon and 4-vinylguaiacol, which were reported for the first time in blueberries. The findings of this study indicated that storage conditions strongly affect the character-impact odorants of blueberries and the overall perceived aroma. Storage at a cold temperature of 1 °C (S1), which is a commercial storage temperature for blueberries, caused a loss in the concentration of several key volatiles, which included (*E*,Z)-2,6-nonadienal, 1-octen-3-ol, 1-octen-3-one, and linalool. This loss altered the blueberries’ aroma, as confirmed by sensory comparisons between treatments. As the storage temperatures increased to 10 °C (S2) and then 20 °C (S3), temperatures designed to simulate distribution and marketing, most of the key volatiles increased to concentrations equivalent to or greater than what was observed at harvest (SC). In concert with this, the sensory panel was not able to discern an aroma difference between SC and S2 or S3. The PCA supported the clear separation of S1 from SC and S3. The overall results indicate that the warmer conditions that typically follow cold storage help to recover character-impact odorants that decline in cold storage. The aroma compounds (*E*,*Z*)-2,6-nonadienal, 1-octen-3-ol, 1-octen-3-one, linalool, and eucalyptol could be used as key odorants indicating the aroma quality of blueberry. Further sensory evaluation to confirm the importance of key odorants when combined as a group in the blueberry matrix is needed to fully ascertain their roles in blueberry aroma.

## Figures and Tables

**Table 1:** Levels of soluble solids, total acidity, and firmness of blueberries subjected to different storage conditions.

Physicochemical Properties	Storage Treatments *^a^*			
	SC *^b^*	S1	S2	S3
Soluble solids (Brix)	13.43 ± 0.38 ^A^	13.27 ± 0.06 ^A^	13.43 ± 0.61 ^A^	13.90 ± 0.45 ^A^
Total acidity (%)	0.45 ± 0.06 ^A^	0.41 ± 0.02 ^AB^	0.35 ± 0.01 ^B^	0.36 ± 0.01 ^AB^
Firmness (g/1 mm) *^c^*	179.61 ± 15.24 ^A^	164.19 ± 10.69 ^A^	157.28 ± 9.85 ^A^	127.89 ± 1.39 ^B^

*^a^* Mean ± S.D. from four replications. Values with different capital letters in a row are significantly different (*p* < 0.05). *^b^* SC = control, S1 = 3 weeks 1 °C, S2 = 3 weeks 1 °C + 1 week 10 °C, S3 = 3 weeks 1 °C + 1 week 10 °C + 2 d 20 °C. *^c^* g/1mm deflection.

**Table 2:** Comparison of flavor dilution factors (FDs) of selected potent odorants in blueberries stored under different conditions.

Volatile Compounds	RI		Odor Properties	FD *^a^*								Identification *^b^*
				Wax				HP-5				
				Storage Treatments				Storage Treatments				
	WAX	HP-5		SC *^c^*	S1	S2	S3	SC *^c^*	S1	S2	S3	
2,3-Butanedione	921	<800	Buttery, Dairy, Milky	4	128	ND *^d^*	16	8	32	8	16	RI, MS, O
3-Methylbutanal	930	<800	Fruity, Green, Malty, Pungent	64	128	256	512	32	128	128	256	RI, O
Methyl-2-methylbutyrate	1013	<800	Blueberry, Fruity, Sweet	1024	128	8	64	1024	ND	4	16	RI, O
Methyl-3-methylbutyrate	1022	<800	Blueberry, Fruity, Sweet	1024	128	64	4096	1024	64	32	1024	RI, MS, O
Ethyl-2-methylbutyrate	1054	840	Fruity, Sweet	32	128	ND	32	128	ND	ND	4	RI, O
Ethyl-3-methylbutyrate	1070	848	Fruity, Sweet	4096	8	64	64	1024	4	32	32	RI, MS, O
Hexanal	1083	811	Fruity, Grassy, Green	32	16	16	64	16	8	8	32	RI, MS, O
(*Z*)-3-Hexenal	1145	805	Apple, Berries, Green	4096	1024	16,384	16,384	2048	1024	4096	4096	RI, MS, O
2-Heptanone	1183	901	Green	64	16	16	8	8	4	4	2	RI, O
Eucalyptol	1210	1031	Minty	64	1024	1024	256	128	256	512	256	RI, MS, O
(*E*)-2-Hexenal	1221	853	Cherries, Green, Sweet	256	128	64	32	128	64	32	16	RI, MS, O
1-Octen-3-one	1278	979	Green, Meaty, Mushroom	4096	4096	16,384	16,384	2048	4096	8192	8192	RI, MS, O
(*E*)-3-Hexen-1-ol	1374	867	Garbage, Grassy, Green, Stinky, Woody	64	32	64	128	64	32	64	128	RI, MS, O
(*Z*)-3-Hexen-1-ol	1387	850	Garbage, Grassy, Green, Stinky, Woody	256	128	64	64	128	64	64	64	RI, MS, O
1-Octen-3-ol	1453	977	Green, Mushroom	8192	16,384	1024	8192	1024	2048	1024	4096	RI, MS, O
Methional	1463	910	Fishy, Meaty, Potato	32	64	256	32	64	128	256	32	RI, O
(*E*)-2-Nonenal	1548	1160	Cucumber, Floral	2	1	1	16	2	1	1	2	RI, MS, O
Linalool	1550	1096	Floral	16,384	8192	8192	16,384	1024	256	256	512	RI, MS, O
(*E*,*Z*)-2,6-Nonadienal	1587	1147	Cucumber, Floral, Green	256	16,384	8192	512	256	1024	64	128	RI, MS, O
Phenylacetaldehyde	1643	1040	Floral, Rosy	2	ND	16	2	2	ND	8	2	RI, MS, O
3-Methylbutyric acid	1678	859	Acidity, Cheesy, Sour	32	8	64	4	32	8	32	4	RI, MS, O
*α*-Terpineol	1701	1192	Floral, Sweet	16	8	16	8	8	2	ND	4	RI, MS, O
Citronellol	1778	1242	Citrusy, Floral, Sweet, Orange	512	1024	1024	512	256	512	512	128	RI, MS, O
Nerol	1810	1232	Floral, Rosy, Sweet	16,384	1024	16,384	16,384	8192	1024	8192	8192	RI, MS, O
Damascenone	1814	1387	Honey, Sweet	4	16	1	8	4	8	2	4	RI, MS, O
Guaiacol	1861	1090	Spicy, Phenolic	16	1024	256	128	8	1024	256	128	RI, MS, O
Geraniol	1862	1252	Citrusy, Floral, Rosy	16,384	16,384	256	8192	8192	8192	256	2048	RI, MS, O
Geranyl acetone	1877	1463	Floral	8	128	64	32	4	64	32	16	RI, O
(*E*)-Cinnamaldehyde	2020	1271	Coconut, Dairy, Sweet,	1024	512	128	128	512	256	128	128	RI, MS, O
*m*-Cresol	2027	1082	Phenolic, Rubber	2	ND	ND	128	2	ND	ND	8	RI, MS, O
*p*-Cresol	2035	1079	Phenolic, Rubber	2	ND	ND	32	2	ND	ND	8	RI, MS, O
*γ*-Decalactone	2168	1466	Coconut, Sweet, Peachy	4096	1024	64	128	2048	512	256	512	RI, MS, O
Eugenol	2171	1356	Clove	1024	128	64	512	512	128	64	128	RI, MS, O
4-Vinylguaiacol	2172	1330	Spicy, Phenolic	32	128	1024	128	8	16	64	8	RI, MS, O
Sotolon	2180	1107	Curry, Spicy	32	128	64	64	16	64	16	16	RI, O
(*Z*)-Cinnamyl alcohol	2299	1267	Floral, Rosy	32	128	64	32	32	64	32	8	RI, O
(*Z*)-Isoeugenol	2300	1414	Clove	8	ND	64	16	8	ND	4	2	RI, MS, O
Vanillin	2569	1399	Vanilla	1024	128	1024	128	256	128	1024	128	RI, MS, O

*^a^* FDs determined by AEDA on DB-Wax and HP-5 columns. *^b^* Identification performed on DB-Wax and DB-5 columns: RIs (retention indices), MS (mass spectrum), or odor properties (O) in agreement with authentic reference standards. *^c^* SC = control, S1 = 3 weeks 1 °C, S2 = 3 weeks 1 °C + 1 week 10 °C, S3 = 3 weeks 1 °C + 1 week 10 °C + 2 d 20 °C. *^d^* ND: not detected.

## Data Availability

The original contributions presented in this study are included in the article/Supplementary Material. Further inquiries can be directed to the corresponding author.

## Supplementary Materials

The following supporting information can be downloaded at https://www.mdpi.com/article/10.3390/foods14071244/s1: Table S1: Selected ion (*m*/*z*) and response factors (R*_f_*) used in volatile analysis; Figure S1: Weight loss following storage of blueberries for 3 weeks at 0 °C, then an additional 1 week at 10 °C, followed by 2 days at 20 °C.

## Abbreviations

The following abbreviations are used in this manuscript:

GC-MS	gas chromatography–mass spectrometry
GC-O	gas chromatography–olfactometry
OAVs	odor activity values
ODTs	odor detection thresholds
AEDA	aroma-extracted dilution analysis
SC	control; blueberries soon after harvest
S2	1 °C ± 0.5 °C for 3 weeks
S3	1 °C ± 0.5 °C for 3 weeks and 10 °C ± 0.5 °C for 1 week
S4	10 °C ± 0.5 °C for 1 week and removed to 20 °C ± 0.5 °C for 2 days
RH	relative humidity
SSC	soluble solids concentration
TA	titratable acidity
IRB	Institutional Review Board
DCM	dichloromethane
HCl	hydrochloric acid
NaOH	sodium hydroxide
Na2SO4	sodium sulfate
UHP	ultrahigh purity
GC-FID	GC–flame ionization detection
SAFE	solvent-assisted flavor evaporation
GC-MS-O	gas chromatography–mass spectrometry–olfactometry
AEDA	aroma extract dilution analysis
FD	flavor dilution
MSD	mass-selective detector
RI	retention index
MS	mass spectral
SAFE-GC-MS	solvent-assisted flavor evaporation–gas chromatography–mass spectrometry
SIM	selective ion monitoring
Rf	response factors
HS-SPME-GC-MS	headspace solid-phase microextraction–gas chromatography–mass spectrometry
NaF	sodium fluoride
NaCl	sodium chlorine
PTFE	polytetrafluoroethylene
TOF-MS	time-of-flight mass spectrometer
HS	headspace
IS	internal standard
t	target analyte
PCA	principal component analysis
ANOVA	analysis of variance
SPME-GC-O	solid-phase microextraction–gas chromatography–olfactometry
CoA	coenzyme A
PTV	programmable temperature vaporizer
