# Supplementary material for: Characterization of Changes in Key Odorants in Blueberries During Simulated Commercial Storage and Marketing by Sensory-Directed Flavor Analysis and Determination of Differences in Overall Perceived Aroma
Source: Foods. 2025 Apr 2;14(7):1244. doi: 10.3390/foods14071244 (PMC11988591; doi:10.3390/foods14071244)
Supplement: Supplementary file 1 [file foods-14-01244-s001.zip › foods-3532360-supplementary.pdf]

Table S1

Selected ion (m/z) and response factors (R_f) used in volatile analysis.

Compound	Ion [a]	Labeled internal standard	Ion [a]	R^2 [b]	R_f [c]
Methyl-3-methylbutyrate	74	Methyl 3-methylbutanoate-d_3	77	0.99+	0.86
Ethyl-3-methylbutyrate	88	Ethyl-3-methylbutyrate-d_5	93	0.99+	0.94
(*Z*)-3-Hexenal	69	Hexanal-d_{12}	64	0.99+	0.81
Hexanal	56	Hexanal-d_{12}	64	0.99+	0.93
Eucalyptol	154	Eucalyptol-d_3	157	0.99+	0.76
(*E*)-2-Hexenal	83	Hexanal-d_{12}	64	0.99+	0.60
(*E*)-3-Hexen-1-ol	67	Linalool-d_3	74	0.99+	0.51
(*Z*)-3-Hexen-1-ol	67	Linalool-d_3	74	0.99+	0.52
1-Octen-3-one	70	Linalool-d_3	74	0.99+	0.51
1-Octen-3-ol	72	Linalool-d_3	74	0.99+	1.12
Linalool	71	Linalool-d_3	74	0.99+	1.31
(*E*,*Z*)-2,6-Nonadienal	70	Linalool-d_3	74	0.99+	1.64
3-Methylbutyric acid	60	Linalool-d_3	74	0.99+	0.48
α-Terpineol	136	α-Terpineol-d_3	139	0.99+	0.69
Nerol	69	Linalool-d_3	74	0.99+	0.45
Citronellol	69	Linalool-d_3	74	0.99+	0.22
Geraniol	69	Linalool-d_3	74	0.99+	0.38
Guaiacol	124	2-Phenyl-d_5-ethan-1,1,2,2-d_4-ol	98	0.99+	3.13
(*E*)-Cinnamaldehyde	131	2-Phenyl-d_5-ethan-1,1,2,2-d_4-ol	98	0.99+	2.71
Decalactone	85	2-Phenyl-d_5-ethan-1,1,2,2-d_4-ol	98	0.99+	0.74
Eugenol	164	2-Phenyl-d_5-ethan-1,1,2,2-d_4-ol	98	0.99+	2.33
4-Vinylguaiacol	135	2-Phenyl-d_5-ethan-1,1,2,2-d_4-ol	98	0.99+	1.38
Vanillin	151	2-Phenyl-d_5-ethan-1,1,2,2-d_4-ol	98	0.99+	2.17

[a] Selected ion used for quantitation. [b] Coefficient of determination for calibration plot.
[c] Response factor.

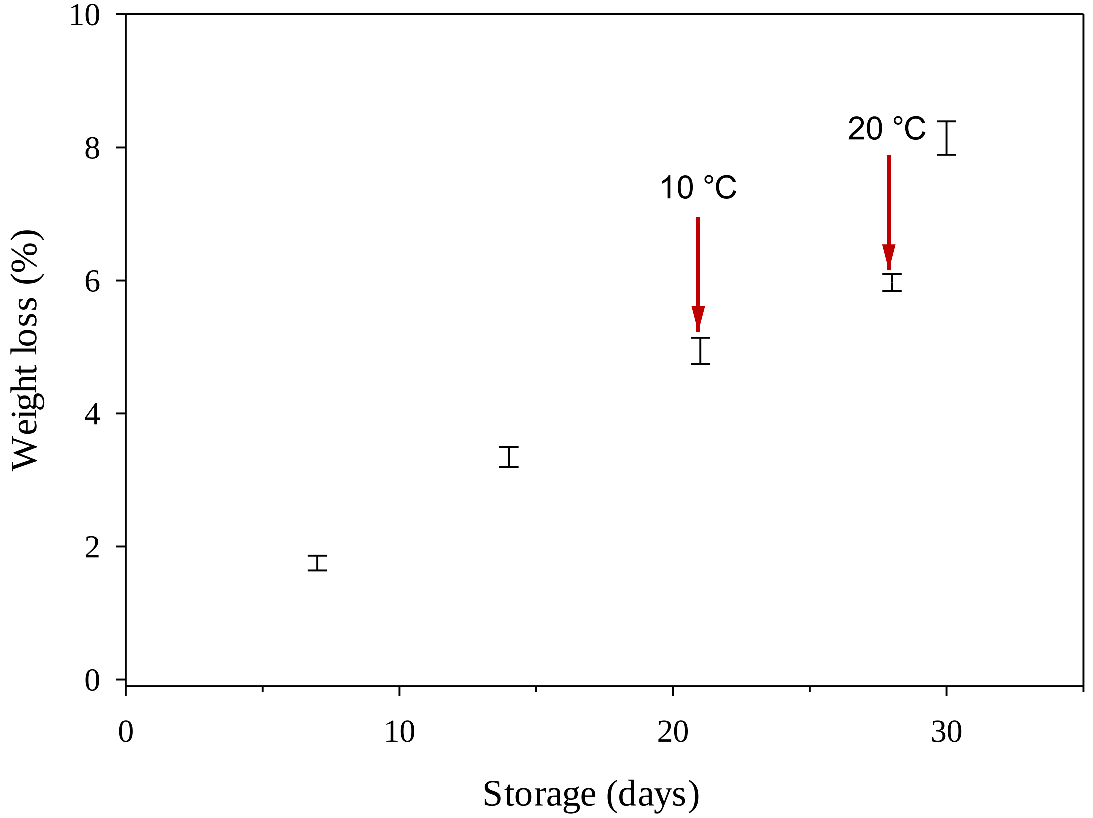

Figure S1. Weight loss following storage of blueberries for 3 weeks at 0 °C, then an additional 1 week at 10 °C, followed by 2 days at 20 °C.
